# Feeding difficulties in typical children, sociodemographic factors, and family perception

**DOI:** 10.1590/2317-1782/e20240160en

**Published:** 2025-02-21

**Authors:** Bruna Franciele Guimarães Trofino, Amélia Augusta de Lima Friche, Denise Brandão de Oliveira e Britto

**Affiliations:** 1 Departamento de Fonoaudiologia, Universidade Federal de Minas Gerais – UFMG - Belo Horizonte (MG), Brasil.

**Keywords:** Feeding Behavior, Sociodemographic Factors, Food Fussiness, Child Nutrition, Speech, Language and Hearing Sciences

## Abstract

**Purpose:**

To verify the association between signs of feeding difficulties in typical children and sociodemographic and economic aspects, parental age and education level, and family perception of feeding difficulties.

**Methods:**

Observational, analytical, cross-sectional study with a probabilistic sample of 113 children aged 2 years to 5 years and 11 months, registered at the Unified Health System in a town in inland Minas Gerais, Brazil. The study applied a sample characterization questionnaire, the Brazilian Child Feeding Scale (EBAI), and the Brazilian Economic Classification Criteria (CCEB) and performed descriptive, univariate, and multivariate analyses with multiple logistic regression.

**Results:**

Children aged 2 and 3 years tended to have more feeding difficulties (p = 0.002) than older children. Preterm children were 3.64 times more likely to have feeding difficulties (p = 0.033) than their peers. Children with signs of feeding difficulties had greater difficulty in food introduction (p = 0.007), ate poorly until 2 years old (p = 0.014), and were 3.7 times more likely to have signs of sensory changes (p = 0.001) than the others.

**Conclusion:**

Children aged 2 and 3 years tend to have more feeding difficulties than older ones. Prematurity, difficulty in introducing foods, and sensory changes are associated with childhood feeding difficulties.

## INTRODUCTION

Children learn to eat through experiences lived during the early years of life, directly influencing food choices in childhood and adulthood^(1,2)^. This process begins in the intrauterine life through nutrition via the umbilical cord, continues after birth with breast milk, and later involves complementary feeding^(3,4)^. Learning to eat is a highly complex process that depends on multiple factors, such as genetic, biological, psychological, sociocultural, environmental, and familial influences, to foster the proper development of healthy eating habits and behaviors^(5)^.

The family plays a significant role from intrauterine nutrition to developing childhood eating habits. A study suggests that foods consumed by the mother during pregnancy are transferred to the fetus (through amniotic fluid) and infant (through breast milk) via odors and flavors^(6)^. During complementary feeding and later in childhood, the family has the crucial role of offering a variety of nutrient-rich foods to help them learn about eating through daily behaviors and food choices.

This process occurs routinely and naturally for most children, shaped by positive and enjoyable experiences. However, 20% to 35% of children experience feeding difficulties during childhood^(7)^. While some present this behavior temporarily, others may experience it for longer and more severely, potentially leading to nutritional deficits and developmental impairments^(1,2,7)^.

Pediatric feeding disorders (PFD) manifest in typically developing children as behaviors like food refusal or meal selectivity^(7-10)^. These behaviors are described as difficulty bringing food to the mouth, difficulty progressing to different textures, eating slowly, showing less interest in food, accepting a limited number of foods or only specific food groups, resisting trying new foods, requiring distractors to increase intake, and having strong food preferences. Children with feeding difficulties may also display behavioral and emotional responses during mealtime, such as refusal, fear, agitation, irritability, and anxiety^(1,7-9)^.

The family is considered a key factor in developing children’s eating habits, as their food choices and prior knowledge about learning to eat impact how the child interacts with and responds to food. Furthermore, how parents or caregivers observe and interpret the children’s signals influences the models and strategies they use to facilitate feeding^(9)^. Families that do not understand the child’s feeding difficulties create unfavorable situations for everyone involved in the feeding process, leading to negative child-food experiences, and reducing their desire to eat. On the other hand, families that model healthy eating behaviors, share enjoyable mealtime experiences, and use positive strategies even when the child has feeding difficulties contribute to the learning process and tend to minimize feeding problems^(11,12)^.

This study aimed to examine the association between signs of feeding difficulties in typically developing children and sociodemographic and economic aspects, parental age and education level, and family perception of feeding difficulties.

## METHODS

This is an observational, analytical, cross-sectional study with a probabilistic sample. Data were collected from children aged 2 years to 5 years and 11 months registered in the public health system of Itaguara, Minas Gerais, Brazil. The study was approved by the Research Ethics Committee of the Federal University of Minas Gerais (UFMG), under CAAE: 53389421.0.0000.5149 and evaluation report: 5.211.897.

The inclusion criteria were typically developing children (as documented in their medical records), aged 2 years to 5 years and 11 months, and whose families signed an informed consent form. The exclusion criteria were children with clinical signs of dysphagia, using alternative feeding methods, with syndromes, genetic malformations, or signs of neurological impairment. Children who did not allow full application of the protocols or whose caregivers did not thoroughly fill out the sample characterization questionnaire, the Brazilian Child Feeding Scale (EBAI, in Portuguese), and the Brazilian Economic Classification Questionnaire (CCEB, in Portuguese) were also excluded.

The inclusion and exclusion criteria were determined based on medical record information provided by the service. Data were collected by applying the sample characterization questionnaire, the EBAI, and the CCEB to the parents.

The sample characterization questionnaire collected identification and clinical data, including medical history, breastfeeding, introduction to complementary feeding, inadequate eating practices, food preferences, sensory aspects, family routine, and family perception of the child’s eating habits. The socioeconomic class was determined through the 2021 CCEB^(13)^, which is based on household assets and income and assesses the purchasing power of Brazilian consumers. The CCEB assigns a score to each asset they own, defining the classes as A1, A2, B1, B2, C, D, and E, according to the sum of these scores – A is the highest, and E is the lowest.

The EBAI^(8)^ is an adapted and validated scale derived from the Montreal Children's Hospital Feeding Scale (MCH-FS)^(14)^, used as a screening tool for childhood feeding difficulties. It has 14 screening items, covering appetite, oral sensory involvement, and oral motor development. The following items reflect parental concerns about the child's general eating, the child’s mealtime behavior, caregivers/feeders’ strategies, and caregivers/feeders' reactions to the child's eating. The scale provides the severity of symptoms and determines the degree of feeding difficulty and the concerns of parents/caregivers. The items’ scores are summed to yield a raw total, which is then compared against a table to determine the total score (T-score). Interpretation classifies scores from 61 to 65 as mild difficulties, 66 to 70 as moderate difficulties, and above 70 as severe difficulties.

Data were collected by the lead researcher in the waiting room of the child health reference service and by community health workers (CHW) during home visits. The lead researcher invited families waiting for a pediatric consultation at the service, whose children fell within the study's age range, to participate in the study. Those who were interested signed an informed consent form and filled out the sample categorization protocols, the EBAI, and the CCEB. The CHWs also distributed the questionnaires and protocols during home visits. The lead researcher trained them regarding the study and how to explain it to the families and invite them to participate. After participants filled out the protocols, they were returned to the researcher for data entry and analysis.

All protocols were analyzed and sorted according to the study’s inclusion and exclusion criteria. Collected data were tabulated in a 2021 Microsoft Office Excel spreadsheet and analyzed using the SPSS – Statistical Package for the Social Sciences, version 21.0. Data underwent descriptive analysis through the frequency distribution of categorical variables.

Pearson's chi-square test and the Mann-Whitney test were used for the association analyses, whose results were considered statistically significant if their p-values were less than 0.05. The Mann-Whitney test was used because the continuous variables “father’s age” and “mother’s age” did not have a normal distribution, as confirmed by the Shapiro-Wilk and Kolmogorov-Smirnov tests, with p-values less than 0.05.

Variables were recategorized as follows: children's age was divided into 1) 2 to 3 years and 2) 4 to 5 years; parents' age was divided into 1) up to 40 years and 2) over 40 years; and education level was categorized as 1) Illiterate/Middle School Incomplete, 2) Middle School Graduate/High School Incomplete, and 3) High School Graduate/Higher Education. The breastfeeding categories were 1) less than 6 months and 2) more than 6 months. The EBAI scale classified children as without difficulties (< 60) and with difficulties (≥ 61), according to the cutoff point proposed in the instrument. Children “with difficulties” included those with mild, moderate, and severe difficulties. The CCEB socioeconomic classes were recategorized as 1) A/B and 2) C/D-E.

Binary logistic regression was performed for the multivariate analysis. Variables with p-values < 0.20 in the univariate analyses were included in the model. The assumptions for using the test were initially checked, including multicollinearity and the absence of outliers. All assumptions were met, as the variance inflation factor (VIF) was less than 10.00, and the tolerance value was greater than 0.1 for all variables. The magnitude of the associations was evaluated using odds ratios (OR) and their respective confidence intervals. The reference categories were Age = 4-5 years; CCEB = C/D-E; Gestational Age = preterm; Introduction to Complementary Feeding = difficult; Discomfort with Noise/Smell/Touch = yes; Feeds in front of a Screen = yes; Feeding Today = poor; Feeding until 2 years = poor.

## RESULTS

Altogether, 113 children participated in the study – 54.9% were females, and 43.3% were 4 years old. The maternal age ranged from 21 to 45 years, with a mean of 36.8 (SD = 5.9) and a median of 33.0. The paternal age ranged from 23 to 60 years, with a mean of 36.8 (SD = 7.2) and a median of 38.0. Moreover, 43.4% of fathers had incomplete middle school, 33.6% of mothers had higher education, and 54.9% of the families belonged to CCEB’s class B2. The sample size varied for some variables due to missing data.

Regarding their medical history, most children were born via cesarean section (71.7%) and were full-term (83.8%). Most mothers reported breastfeeding their children until the 6^th^ month (88.5%), and more than half of the mothers introduced complementary feeding at 6 months (59.3%). Also, 82.3% of mothers stated that their children ate well until 2 years old, although 69.9% reported difficulties introducing foods. As for sensory aspects, 70.5% reported that their children were not bothered by noise, smell, touch, or textures (Table 1).

**Table 1:** Descriptive analysis of clinical data

**Variables**	**N**	**%**
Delivery		
Normal	32	28.3
Cesarean	81	71.7
Total	113	100.0
Gestational age		
Full term	94	83.2
Preterm	19	16.8
Total	113	100.0
Breastfeeding		
No	13	11.5
Yes	100	88.5
Total	113	100.0
Age at food introduction		
Before 6 months	46	40.7
After 6 months	67	59.3
Total	113	100.0
Food introduction		
Easy	34	30.1
Difficult	79	69.9
Total	113	100.0
Feeding up to 2 years old		
Ate well	93	82.3
Ate poorly	20	17.7
Total	113	100.0
Bothered by noise/smell/touch		
No	79	70.5
Yes	33	29.5
Total	112	100.0

Caption: N = number of individuals

The analysis of eating behavior data shows that most children (68.1%) were classified as having no feeding difficulties, and 54.4% of parents perceived that their children ate well. Most children ate at the table (61.1%), in the presence of companions other than their parents (61.9%). Most children (69.0%) ate in front of the TV, tablet, or mobile phone. The caregivers considered this habit detrimental but reported that it helped their children eat better (61.1%) (Table 2).

**Table 2:** Descriptive analysis of EBAI data and family perception of feeding

**Variables**	**N**	**%**
EBAI		
No difficulties	77	68.1
Mild difficulty	27	23.9
Moderate difficulty	7	6.2
Severe difficulty	2	1.8
Total	113	100.0
Mealtime companion		
Mother	43	38.1
Others	70	61.9
Total	113	100.0
How the family eats		
Together	71	62.8
Separately	42	37.2
Total	113	100.0
How the child eats		
At the table with the family	69	61.1
Somewhere else	44	38.9
Total	113	100.0
The child watches TV, tablet, mobile phone during meals		
No	35	31.0
Yes	78	69.0
Total	113	100.0
When they watch TV, tablet, mobile phone during meals		
They eat less food amount and variety	11	14.1
They eat the same food amount and variety	35	44.9
They eat a greater food amount and variety	32	41.0
Total	78	100.0
Do you consider it harmful to watch TV, tablet, or mobile phone during meals?		
Yes, I do not allow it	31	27.4
Yes, but I allow it so they can eat well	69	61.1
No	13	11.5
Total	113	100.0
How do you perceive your child’s current eating?		
Very good	21	18.8
Good	61	54.4
Poor	24	21.4
Very poor	6	5.4
Total	112	100.0
		

Caption: N = number of individuals; EBAI = Brazilian Child Feeding Scale

After analyzing the descriptive data, an association analysis was conducted between the children’s signs of feeding difficulties and sociodemographic data. It was observed that children aged 4 to 5 years tended not to show signs of feeding difficulties. The other associations were not statistically significant (Table 3).

**Table 3:** Association analysis between feeding difficulty (EBAI) and sociodemographic data

**Variables**	**EBAI**		
	**No difficulties**	**With difficulties**	**p-value**
	**N (%)**	**N (%)**	
Sex			
Females	45 (58.4)	17 (47.2)	0.264
Males	32 (41.6)	19 (52.8)	
Total	77 (100.0)	36 (100.0)	
Age			
2-3 years	9 (11.7)	13 (36.1)	0.002*
4-5 years	68 (88.3)	23 (53.9)	
Total	77 (100.0)	36 (100.0)	
Paternal age			
Up to 40 years	54 (70.1)	21 (58.3)	0.226
Over 40 years	23 (29.9)	15 (41.7)	
Total	77 (100.0)	36 (100.0)	
Paternal education level			
Illiterate/High School Incomplete	41 (53.2)	18 (50.0)	0.748
High School Graduate/Higher Education	36 (46.8)	18 (50.0)	
Total	77 (100.0)	36 (100.0)	
Maternal age			
Up to 40 years	68 (88.3)	30 (83.3)	0.467
Over 40 years	9 (11.7)	6 (16.7)	
Total	77 (100.0)	36 (100.0)	
Maternal education level			
Illiterate/High School Incomplete	23 (29.9)	10 (27.8)	0.820
High School Graduate/Higher Education	54 (70.1)	26 (72.2)	
Total	77 (100.0)	36 (100.0)	
CCEB			
A/B	55 (71.4)	30 (80.3)	0.172
C/D-E	22 (28.6)	6 (16.7)	
Total	77 (100.0)	36 (100.0)	

Pearson’s chi-square test

* p-value ≤ 0.05

Caption: N = number of individuals; EBAI = Brazilian Child Feeding Scale; CCEB = Brazilian Economic Classification Criteria

The medical history survey showed a higher proportion of preterm children among those with signs of feeding difficulties (p = 0.033) than those with no difficulties. The introduction to complementary feeding was more challenging to children with signs of feeding difficulties (p = 0.007) than the others. Also, poor feeding until 2 years old was associated with feeding difficulties (p = 0.014), and a higher proportion of children with sensory signs (discomfort with noise/smell/touch) were found among those with feeding difficulties (p = 0.001) (Table 4).

**Table 4:** Association analysis between feeding difficulty (EBAI), clinical data, and family perception of feeding

**Variables**	**EBAI**		
	**No difficulties**	**With difficulties**	**p-value**
	**N (%)**	**N (%)**	
Delivery			
Normal	21 (27.3)	11 (30.6)	0.718
Cesarean	56 (72.7)	25 (69.4)	
Total	77 (100.0)	36 (100.0)	
Gestational age			
Full term	68 (88.3)	26 (72.2)	0.033*
Preterm	9 (11.7)	10 (27.8)	
Total	77 (100.0)	36 (100.0)	
Breastfeeding			
Less than 6 months	8 (10.4)	5 (13.9)	0.587
More than 6 months	69 (89.6)	31 (86.1)	
Total	77 (100.0)	36 (100.0)	
Food introduction			
Before 6 months	33 (42.9)	13 (36.1)	0.496
After 6 months	44 (57.1)	23 (63.9)	
Total	77 (100.0)	36 (100.0)	
How was the food introduction			
Easy	17 (22.1)	17 (47.2)	0.007*
Difficult	60 (77.9)	19 (52.8)	
Total	77 (100.0)	36 (100.0)	
Feeding up to 2 years old			
Ate well	68 (88.3)	25 (69.4)	0.014*
Ate poorly	9 (11.7)	11 (30.6)	
Total	77 (100.0)	36 (100.0)	
Bothered by noise/smell/touch			
No	61 (80.3)	18 (50.0)	0.001*
Yes	15 (19.7)	18 (50.0)	
Total	77 (100.0)	36 (100.0)	
Mealtime companion			
Mother	28 (36.4)	15 (41.7)	0.589
Others	49 (63.6)	21 (58.3)	
Total	77 (100.0)	36 (100.0)	
How the family eats			
Together	50 (64.9)	21 (58.3)	0.499
Separately	27 (35.1)	15 (41.7)	
Total	77 (100.0)	36 (100.0)	
How the child eats			
At the table with the family	47 (61.0)	22 (61.1)	0.994
Somewhere else	30 (39.0)	14 (38.9)	
Total	77 (100.0)	36 (100.0)	
The child watches TV/tablet/mobile phone during meals			
No	27 (35.1)	8 (22.2)	0.169
Yes	50 (64.9)	28 (77.8)	
Total	77 (100.0)	36 (100.0)	
Do you consider it harmful to watch TV, tablet, or mobile phone during meals?			
Yes, I do not allow it	23 (29.9)	8 (22.2)	0.653
Yes, but I allow it so they can eat well	46 (59.7)	23 (63.9)	
No	8 (10.4)	5 (13.9)	
Total	77 (100.0)	36 (100.0)	
How do you perceive your child’s current eating?			
Good	59 (77.6)	23 (63.9)	0.125
Poor	17 (22.4)	13 (36.1)	
Total	76 (100.0)	36 (100.0)	

Pearson’s chi-square test

* p-value ≤ 0.05

Caption: N = number of individuals; EBAI = Brazilian Child Feeding Scale

Table 5 presents the initial and final models of the multivariate analysis of the EBAI with sociodemographic and clinical data, using binary logistic regression. The analysis shows that “Age” remained in the final model with significant values – 0.32 OR (68% lower), indicating that children aged 4 to 5 years had a lower likelihood of abnormal EBAI results than children aged 2 to 3 years. Premature children had 3.4 times higher odds of having abnormal EBAI results than full-term ones, and children who reported discomfort with noise, smell, or touch had 3.7 times higher odds of having abnormal EBAI results than those who did not experience these discomforts.

**Table 5:** Multivariate analysis of binary logistic regression between EBAI and age, clinical data, and family perception of eating

**Variables**	**Abnormal EBAI Result**					
	**Initial model**			**Final model**		
	**OR**	**CI**	**p-value**	**OR**	**CI**	**p-value**
Age	0.250	0.075-0.829	0.023	0.323	0.078 – 0.693	0.009*
CCEB	0.490	0.135-1.782	0.279	---	---	---
Gestational age	3.210	0.847-12.1673	0.086	3.415	1.067 – 10.931	0.039*
Food introduction	0.695	0.209-2.313	0.695	---	---	---
Discomfort with noise/smell/touch	2.229	0.675-7.366	0.189	3.743	1.465 – 9.565	0.006*
Mealtime screen exposure	1.621	0.538-4.882	0.391	---	---	---
Current feeding	1.214	0.397-3.710	0.734	---	---	---
Feeding up to 2 years old	1.501	0.350 – 6.440	0.585	---	---	---

Wald test, stepwise model

* p-value ≤ 0.05

Caption: OR = odds ratio; CI = confidence interval; EBAI = Brazilian Child Feeding Scale. **Reference variables:** Age = 4-5 years; CCEB = C/D-E; Gestational age = preterm; Food introduction = difficult; Discomfort with noise/smell/touch = yes; Mealtime screen exposure = yes; Current feeding = poor; Feeding up to 2 years old = poor

## DISCUSSION

This study examined the association between signs of feeding difficulties in children aged 2 to 5 years and sociodemographic and socioeconomic data, medical history, parental education, and family perception of the child’s feeding difficulties. Children aged 2 to 3 years, born preterm, and with discomfort related to noise, smell, or touch had higher odds of experiencing feeding difficulties.

The study children had a 31.9% prevalence of feeding difficulties, a value similar to that found in other studies in the literature. For instance, a Canadian study^(15)^ found that 30% of children aged 2 and a half to 4 and a half years were characterized as picky eaters. Another study that followed children aged 3 to 11 years reported that 16 to 22% of children had feeding difficulties regardless of age, and 39% of children were classified as picky eaters at some point during the study^(16)^.

There was a trend for children aged 2 and 3 years to have more feeding difficulties than the other age groups, possibly because children in this age range are developing autonomy and seeking more independence during mealtime. As a result, they begin to choose foods based on their preferences, avoid unfamiliar foods, or trigger feelings of refusal and/or food aversion. Some authors report that these behaviors tend to decrease with age^(17)^, while other studies suggest that the prevalence of feeding difficulties remains stable from 2 and a half to 4 and a half years old^(16)^. Another study made three assessments from 1 and a half years to 6 years old and found that the prevalence of difficulties was 26.5% at 1 and a half years, increasing to 27.6% at 3 years, and declining to 13.2% at 6 years old^(18)^. This corroborates other findings^(16,17)^ that show a peak in feeding difficulties at 3 years old.

Concerning risk factors for childhood feeding difficulties, the present study found that preterm children had 3.64 times higher odds of developing signs of feeding difficulties than full-term children. It is well known that prematurity, besides triggering potential physical and psychosocial difficulties for the infant, also increases the risk of developing feeding behavior disorders^(19-21)^.

Migraine’s study^(19)^ compared two cohorts, one with full-term children and the other with preterm children. It found that preterm children scored worse on the drive to eat and had lower scores on their food repertoire. Another study reports that preterm infants were at higher risk for food refusal/picky eating^(22)^. These two studies included preterm infants with associated comorbidities – unlike the present one, which excluded infants with associated comorbidities from the sample.

It is important to emphasize that prematurity is a risk factor that should not be overlooked, even in healthy children, as it may be associated with selective eating and behavioral mealtime problems, as found in this study. Such behaviors may be related to oral dysfunctions, early interruption or absence of breastfeeding, early introduction of complementary feeding, and neurobehavioral aspects^(23)^. Furthermore, parents of preterm children often experience significant concern and anxiety when feeding their children, and these feelings can negatively influence mealtime eating behavior^(22,24)^.

The present study also found signs of difficulties after 2 years old and in introducing complementary foods. Introducing solid foods is quite challenging for families, and their prior knowledge directly impacts how they will begin the introduction of foods and handle the challenges during the process. Early introduction of complementary feeding (before 6 months old) can trigger various health issues for the baby and negatively influence their learning to eat because they will not have developed all the necessary signs of feeding readiness^(25,26)^.

The literature reports that one in every four babies is reluctant to the introduction of new textures and flavors^(4)^. This agrees with a study^(27)^ that reported that children characterized as problematic eaters had difficulties with breastfeeding and the introduction of solid foods and continued to face such challenges into childhood. Infants must have sensory and taste experiences as complementary foods are introduced to expand their taste and develop a broader food repertoire and positive food experiences^(1)^.

Another significant factor is repeatedly offering foods the baby has previously rejected, which should be done six to 15 times for the child to learn to accept certain foods^(12)^. It is inferred that families who have not received information about proper feeding practices may trigger negative behaviors and experiences during the child’s early years, which can persist throughout childhood.

Signs of feeding difficulties were not associated with the parents' socioeconomic factors. However, the literature reports that older parents and those with a higher socioeconomic status and a higher level of education tend to make better food choices and provide healthier meals^(19,28)^. Other studies indicate that selective eating was more common in children from low-income families, and their parents tended to be younger than those of non-picky eaters^(18)^. It is inferred that sociodemographic factors are related to food quality – although this alone does not guarantee successful learning to eat. Other factors, such as cultural, social, and behavioral aspects, also play a role in shaping children's eating habits.

According to the literature, feeding is also directly related to the person's multisensory experiences. Hence, consuming foods with varied forms, textures, flavors, and smells can be aversive for those with sensory sensitivity^(29)^. This study found that children with signs of sensory risk had more feeding difficulties than others, agreeing with studies that show an association between food rejection and the ability to perceive subtle sensory changes in foods^(17,23,30)^.

Signs of feeding difficulties were not associated with screen exposure during meals, but 69% of the children in the sample ate while watching screens. Among these children who ate while exposed to screens, 61.1% of the families reportedly believed it is harmful, but they allowed it because it helped the children eat better. The literature includes studies that link excessive screen time to negative child health outcomes, such as language delays, attention difficulties, cognitive delays, and feeding problems^(15,25)^.

Bahadur's study^(25)^ found that only children with signs of feeding difficulties had longer screen exposure. Other studies link TV exposure to feeding disorders and lower consumption of fruits and vegetables^(15,28)^. No studies were found specifically relating screen exposure to food refusal and selectivity behaviors. It is inferred that families allow this habit to help their children eat more and consume foods they typically reject while being entertained by screens. However, having children eat while distracted by screens can affect their regulated hunger and satiety perception and hinder their ability to perceive texture, flavor, and consistency, ultimately impairing their learning to eat^(15,23)^.

This study is innovative regarding EBAI use, which has been recently translated and validated for use in Brazil. However, it has some limitations that should be considered. The questionnaires used in the research – sample characterization questionnaire, CCEB, and EBAI – were filled out by the families. Therefore, difficulty and normality were subjective concepts, depending on each participant's perception. There was also an uneven age distribution in the study, as many children were aged 4 and 5 years and few were 2 and 3 years. Thus, further studies are needed with more homogeneous samples in terms of age and different settings for more reliable results.

## CONCLUSION

Young children, aged 2 and 3 years, had a greater tendency to show signs of feeding difficulties. Furthermore, a statistically significant association was found with prematurity, difficulties in introducing food and in the second year of life, and signs of sensory changes.

These results provide greatly important information for healthcare professionals working with babies and children and can support more specific and targeted guidance on feeding difficulties, helping implement preventive actions.
