# Supplementary material for: Feeding difficulties in typical children, sociodemographic factors, and family perception
Source: Codas. 2025 Feb 21;37(2):e20240160. doi: 10.1590/2317-1782/e20240160en (PMC11864763; doi:10.1590/2317-1782/e20240160en)
Supplement: Translation

# Translation: Dificuldade alimentar em crianças típicas, fatores sociodemográficos e percepção da família

**DOI:** 10.1590/2317-1782/e20240160pt

**Authors:** Bruna Franciele Guimarães Trofino, Amélia Augusta de Lima Friche, Denise Brandão de Oliveira e Britto

**Affiliations:** 1 Departamento de Fonoaudiologia, Universidade Federal de Minas Gerais – UFMG - Belo Horizonte (MG), Brasil.

**Keywords:** Comportamento Alimentar, Fatores Sociodemográficos, Seletividade Alimentar, Nutrição Infantil, Fonoaudiologia

## Abstract

**Objetivo:**

Verificar a associação entre sinais de dificuldade alimentar em crianças típicas e aspectos sociodemográficos, econômicos, idade e escolaridade dos pais e percepção da família sobre as dificuldades alimentares.

**Método:**

Estudo observacional, analítico, transversal com amostra probabilística. Participaram 113 crianças de 2 anos a 5 anos e 11 meses cadastradas no SUS de cidade do interior do estado. Foram aplicados questionário de amostra, Escala Brasileira de Alimentação Infantil (EBAI) e Critério de Classificação Econômica Brasil (CCEB). Realizadas análises descritiva, uni e multivariada, por meio de regressão logística múltipla.

**Resultados:**

Foi observada a tendência de crianças com 2 e 3 anos apresentarem maior dificuldade para se alimentar (p=0,002) quando comparadas às crianças mais velhas. Crianças pré-termo tiveram 3,64 mais chances de apresentar dificuldades alimentares (p=0,033) do que seus pares. Verificou-se que crianças com sinais de dificuldades alimentares demonstraram maior dificuldade no processo de introdução alimentar (p=0,007), se alimentaram mal até os dois anos (p=0,014) e apresentam 3,7 mais chances de apresentar sinais de alterações sensoriais (p=0,001) do que as demais.

**Conclusão:**

Crianças de 2 e 3 anos demonstram tendência de apresentar mais dificuldades alimentares que as crianças maiores. A prematuridade, dificuldade na introdução alimentar e alterações sensoriais são fatores associados a dificuldade alimentar na infância.

## INTRODUÇÃO

O aprendizado do comer é construído a partir de experiências vivenciadas nos primeiros anos de vida, que influenciam diretamente as escolhas alimentares na infância e vida adulta^(1,2)^. Esse processo inicia-se no período intrauterino pela nutrição via cordão umbilical, após o nascimento pelo leite materno e posteriormente pela alimentação complementar^(3,4)^. O processo de aprendizado do comer é bastante complexo e depende de fatores múltiplos como: fatores genéticos, biológicos, psicológicos, socioculturais, ambientais e familiares para que promova adequado desenvolvimento de hábitos e comportamentos alimentares saudáveis^(5)^.

Desde a nutrição intrauterina até a construção de hábitos alimentares durante a infância, a família tem importância significativa. Estudo descreve a possibilidade de que os alimentos consumidos pela mãe durante a gestação, por meio do líquido amniótico e período de lactação por meio do leite materno, são transferidos para o feto e/ou bebê por meio de odores e sabores^(6)^. No período de introdução alimentar, e posteriormente na infância, a família tem a importante função de oferecer alimentos variados ricos em nutrientes e fornecer o aprendizado do comer por meio dos comportamentos e escolhas alimentares diárias.

Para a maioria das crianças, esse processo ocorre de forma rotineira e natural, construídos por experiências positivas e prazerosas. Porém, entre 20 e 35% das crianças apresentam dificuldades alimentares na infância^(7)^. Embora algumas crianças apresentem esse comportamento de forma transitória, outras podem apresentar por longo período de tempo e de forma mais grave, podendo gerar prejuízos nutricionais e no seu desenvolvimento^(1,2,7)^.

Os distúrbios alimentares pediátricos (DAP) se manifestam em crianças típicas como comportamentos de recusa ou seletividade alimentar durante a refeição^(7-10)^. Esses comportamentos são descritos como: dificuldade em levar alimentos até a boca, dificuldade em avançar em texturas diferentes, comer lentamente, apresentar menos interesse pela comida, aceitar um número reduzido de alimentos ou aceitar somente grupos de alimentos específicos, resistência em provar novos alimentos, necessidade de usos de distratores para aumentar a ingestão e apresentar fortes preferências alimentares. Além disso, as crianças com dificuldades alimentares podem apresentar respostas comportamentais e emocionais no momento das refeições, como recusa, medo, agitação, irritabilidade ou ansiedade^(1,7-9)^.

A família é considerada peça chave para o desenvolvimento dos hábitos alimentares desenvolvidos pela criança, pois as escolhas alimentares e o conhecimento prévio sobre o aprendizado do comer impactam a forma como a criança interage e se comporta com os alimentos. Além disso, a forma como os pais ou cuidadores observam e interpretam os sinais fornecidos pela criança, influencia em quais modelos e estratégias irão utilizar para facilitar a alimentação^(9)^. Famílias que não compreendem as dificuldades alimentares da criança, criam situações desfavoráveis entre todos os envolvidos no processo de alimentação, com isso, fornecem experiências negativas entre o alimento e o desejo de comer. Contudo, famílias que fornecem um modelo de alimentação saudável, que compartilham do momento da refeição de forma prazerosa e que fornecem estratégias positivas mesmo na presença de dificuldades alimentares, contribuem para o aprendizado e tendem a minimizar problemas relacionados a alimentação^(11,12)^.

O objetivo desse estudo foi verificar a associação entre sinais de dificuldade alimentar em crianças típicas e aspectos sociodemográficos, econômicos, idade e nível de escolaridade dos pais e percepção da família sobre as dificuldades alimentares.

## MÉTODO

Esse estudo caracteriza-se como observacional analítico de delineamento transversal com amostra probabilística. Foi realizada coleta de dados de crianças de 2 anos a 5 anos e 11 meses cadastradas no sistema único de saúde da cidade de Itaguara, Minas Gerais, Brasil. Esse estudo foi aprovado pelo Comitê de Ética em Pesquisa da Universidade Federal de Minas Gerais (UFMG) CAAE: 53389421.0.0000.5149, sob o parecer: 5.211.897.

Os critérios de inclusão foram crianças na faixa etária de 2 a 5 anos e 11 meses, que apresentassem desenvolvimento típico relatado no prontuário médico e cujas famílias assinaram o Termo de Consentimento Livre e Esclarecido (TCLE). Foram excluídas as crianças que apresentaram sinais clínicos de disfagia, que faziam uso de via alternativa de alimentação, síndromes, malformações genéticas e sinais de comprometimento neurológico. Também foram excluídas crianças que não permitiram a aplicação dos protocolos por completo e crianças cujos responsáveis não responderam na íntegra o questionário de caracterização da amostra, a Escala Brasileira de Alimentação Infantil (EBAI) e o questionário de classificação econômica Brasil (CCEB).

Os critérios de inclusão e exclusão foram obtidos por meio das informações descritas nos prontuários fornecidos pelo serviço. Os dados foram obtidos a partir da aplicação do questionário de caracterização da amostra, da escala EBAI e do questionário de classificação econômica Brasil (CCEB) preenchidos pelos pais.

No questionário de caracterização da amostra foram coletados dados de identificação, clínicos - história pregressa, amamentação, introdução alimentar, práticas alimentares inadequadas, preferências alimentares, aspectos sensoriais, rotina familiar e percepção da família acerca da alimentação da criança. A classe socioeconômica, foi obtida pelo Critério de Classificação Econômica Brasil (CCEB 2021)^(13)^ que é uma classificação baseada nos bens e renda do domicílio e que avalia o poder de compra dos consumidores brasileiro. Para cada bem possuído há uma pontuação e cada classe é definida pela soma dessa pontuação. As classes definidas pelo CCEB são A1, A2, B1, B2, C, D e E, sendo a A a classe mais alta e E a mais baixa.

A Escala Brasileira de Alimentação Infantil EBAI^(8)^ é uma escala adaptada, validada e oriunda da Escala de Alimentação do Hospital Pediátrico de Montreal (Montreal Children´s Hospital Feeding Scale – MCH-FS)^(14)^, utilizada como instrumento de rastreio para dificuldades alimentares na infância. A escala é composta de 14 itens de rastreio, entre eles: apetite, envolvimento sensorial oral e desenvolvimento motor oral; os seguintes itens refletem preocupações parentais sobre a alimentação da criança em geral, comportamento da criança na hora da refeição, estratégias usadas pelos cuidadores/alimentadores, e reações dos cuidadores/alimentadores em relação à alimentação da criança. A escala fornece a gravidade dos sintomas apresentados e determina o grau da dificuldade alimentar e das preocupações dos pais/cuidadores. As pontuações das questões são somadas chegando em um total bruto, que é colocado em uma tabela para verificar o score total (T-score). A interpretação classifica pontuações de 61 a 65 como dificuldades leves, 66 a 70 dificuldades moderadas, e acima de 70 como dificuldades graves.

A coleta de dados foi realizada pela pesquisadora principal, na sala de espera do serviço de referência à saúde da criança e pelas Agentes Comunitárias de Saúde (ACS) em visitas domiciliares. As famílias que estavam presentes na sala de espera do serviço, aguardando consulta com a médica pediatra, cujas crianças enquadravam-se na faixa etária da pesquisa, foram convidadas a participar da pesquisa. As que manifestaram interesse assinaram o TCLE, preencheram os protocolos de categorização de amostra, a Escala EBAI e o CCEB. As ACSs também realizaram a entrega dos questionários e protocolos, durante as visitas domiciliares. As ACSs receberam capacitação pela pesquisadora principal sobre a pesquisa e como deveriam explicar e convidar as famílias a participarem. Após o preenchimento, os protocolos foram devolvidos a pesquisadora, para tabulação e análise.

Todos os protocolos foram analisados e separados de acordo com os critérios de inclusão e exclusão definidos no estudo. Os dados coletados foram tabulados em planilha do Microsoft Office Excel - versão 2021 e analisados no programa SPSS – Statistical Package for the Social Sciences Incorporation 53 – versão 21.0. Foi realizada análise descritiva dos dados, por meio da distribuição de frequência das variáveis categóricas.

Para as análises de associação foram utilizados os testes Qui-quadrado de Pearson e Mann-Whitney, sendo considerados como significantes os que apresentaram valor de p<0,05. A escolha do último teste deve-se ao fato das variáveis contínuas “idade do pai” e “idade da mãe” não apresentarem distribuição normal, confirmada por meio dos testes dos testes Shapiro Wilk e Kolmogorov-Smirnov, cujos valores-p encontrados foram menores que 0,05.

Foi realizada a recategorização de variáveis, sendo: a idade das crianças em 1) de 2 a 3 anos e 2) 4 a 5 anos; a idade dos pais em: 1) até 40 anos e 2) mais de 40 anos e escolaridade em: 1) Analfabeto/Fundamental Incompleto, 2) Fundamental completo/Médio incompleto e 3) Médio completo/Superior. Em relação a amamentação, foi considerado 1) período menor do que seis meses e 2) maior que seis meses. Quanto a Escala EBAI, as crianças foram classificadas como sem dificuldades (<60) e com dificuldades (≥61), segundo o ponto de corte proposto no instrumento e no aspecto com dificuldades, foram incluídas crianças que apresentavam dificuldades leves, moderadas e graves. No questionário CCEB, as classes foram recategorizadas como 1) A/B e 2) C/D-E.

Para a análise multivariada, realizou-se regressão logística binária. Dessa forma, entraram no modelo as variáveis com valores-p<0,20 nas análises univariadas. Inicialmente, foram analisados os pressupostos para utilização do teste, ou seja, o de multicolineariedade e ausência de *outliers*. Todos os pressupostos foram cumpridos, visto que o valor do fator de inflação da variância (VIF) foi menor que 10,00 e o de tolerância maior que 0,1 para todas as variáveis. As magnitudes das associações foram avaliadas pelas razões de chances (odds *ratio*) e seus respectivos intervalos de confiança. As categorias de referência foram: Idade= 4-5 anos; CCEB= C/D-E; Idade gestacional= pré-termo; Introdução alimentar= difícil; Incômodo barulho/cheiro/toque= sim; alimenta em frente a tela= sim; como alimenta hoje= mal; alimentação até dois anos= comia mal.

## RESULTADOS

Participaram do estudo 113 crianças, sendo 54,9% do sexo feminino e 43,3% das crianças tinham idade de 4 anos. A idade materna variou de 21 a 45 anos, com a média de 36,8 (DP=5,9) e mediana 33,0 e idade paterna variou entre 23 a 60 anos, com média de 36,8 (DP=7,2) e mediana de 38,0. Em relação ao nível de escolaridade dos pais, 43,4% dos pais possuíam ensino fundamental incompleto e 33,6% das mães possuíam ensino superior e 54,9% das famílias pertenciam a classe B2 no CCEB. Houve variação do N em algumas variáveis em decorrência de dados faltantes.

Em relação aos dados da história pregressa, a maioria das crianças nasceu de parto cesáreo (71,7%) e a termo (83,8%). A maioria das mães relatou que amamentaram seus filhos até o sexto mês (88,5%), mais da metade das mães realizaram a introdução alimentar aos seis meses (59,3%). Do total de mães, 82,3% respondeu que até os dois anos seus filhos se alimentavam bem, embora 69,9% relatou dificuldades no processo de introdução de alimentos. Considerando os aspectos sensoriais, 70,5% relatou que os filhos não se incomodavam com barulho, cheiro, toque ou texturas (Tabela 1).

**Tabela 1:** Análise descritiva dos dados clínicos

**Variáveis**	**N**	**%**
**Parto**		
Normal	32	28,3
Cesárea	81	71,7
Total	113	100,0
**Idade gestacional**		
Termo	94	83,2
Pré-termo	19	16,8
Total	113	100,0
**Amamentação**		
Não	13	11,5
Sim	100	88,5
Total	113	100,0
**Idade introdução alimentar**		
Antes 6 meses	46	40,7
Após 6 meses	67	59,3
Total	113	100,0
**Introdução alimentar**		
Fácil	34	30,1
Difícil	79	69,9
Total	113	100,0
**Alimentação até dois anos**		
Comia bem	93	82,3
Comia mal	20	17,7
Total	113	100,0
**Incomoda-se com barulho‎/cheiro‎/toque**		
Não	79	70,5
Sim	33	29,5
Total	112	100,0

Legenda: N = número de indivíduos

A análise dos dados referentes ao comportamento alimentar demonstra que a maioria das crianças, 68,1%, foi classificada como sem dificuldades alimentares e 54,4% dos pais relataram que na sua percepção os filhos se alimentam bem. A maioria das crianças se alimenta na mesa (61,1%), na presença de outros acompanhantes sem ser os próprios pais (61,9%). A maioria das crianças (69,0%) estudadas, se alimenta em frente à TV, *tablet* ou celular. Embora os responsáveis achem esse hábito prejudicial, relatam que contribuem para que as crianças se alimentem melhor (61,1%) (Tabela 2).

**Tabela 2:** Análise descritiva dos dados referentes escala EBAI e percepção da família acerca da alimentação

**Variáveis**	**N**	**%**
**EBAI**		
Sem dificuldades	77	68,1
Dificuldade leve	27	23,9
Dificuldade moderada	7	6,2
Dificuldade grave	2	1,8
Total	113	100,0
**Acompanhante durante as refeições**		
Mãe	43	38,1
Outros	70	61,9
Total	113	100,0
**Como a família se alimenta**		
Juntos	71	62,8
Separados	42	37,2
Total	113	100,0
**A criança se alimenta**		
Na mesa com a família	69	61,1
Em outro local	44	38,9
Total	113	100,0
**A criança se alimenta assistindo TV, tablet, celular**		
Não	35	31,0
Sim	78	69,0
Total	113	100,0
**Quando se alimenta assistindo TV, tablet, celular**		
Come menos quantidade e variedade de alimentos	11	14,1
Come a mesma quantidade e variedade de alimentos	35	44,9
Come mais quantidade e variedade de alimentos	32	41,0
Total	78	100,0
**Acha prejudicial alimentar em frente TV, tablet, celular**		
Sim, não permito	31	27,4
Sim, mas permito para que coma bem	69	61,1
Não	13	11,5
Total	113	100,0
**Hoje, como considera como seu filho se alimenta**		
Muito bem	21	18,8
Bem	61	54,4
Mal	24	21,4
Muito mal	6	5,4
Total	112	100,0
		

Legenda: N = número de indivíduos; EBAI = Escala Brasileira de Alimentação Infantil

Após a análise dos dados descritivos, foi realizada a análise de associação entre sinais de dificuldade alimentar das crianças e os dados sociodemográficos. Foi observado que há uma tendência em crianças com idade entre quatro e cinco anos não apresentarem sinais de dificuldade para se alimentar. As demais associações não apresentaram significância estatística (Tabela 3).

**Tabela 3:** Análise de associação entre dificuldade para alimentar (EBAI) e dados sociodemográficos

**Variáveis**	**EBAI**		
	**Sem dificuldade**	**Com dificuldade**	**valor-p**
	**N (%)**	**N (%)**	
**Sexo**			
Feminino	45 (58,4)	17 (47,2)	0,264
Masculino	32 (41,6)	19 (52,8)	
Total	77 (100,0)	36 (100,0)	
**Idade**			
2-3 anos	9 (11,7)	13 (36,1)	0,002*
4-5 anos	68 (88,3)	23 (53,9)	
Total	77 (100,0)	36 (100,0)	
**Idade paterna**			
Até 40 anos	54 (70,1)	21 (58,3)	0,226
Acima de 40 anos	23 (29,9)	15 (41,7)	
Total	77 (100,0)	36 (100,0)	
**Escolaridade paterna**			
Analfabeto/Médio Incompleto	41 (53,2)	18 (50,0)	0,748
Médio Completo/Superior	36 (46,8)	18 (50,0)	
Total	77 (100,0)	36 (100,0)	
**Idade materna**			
Até 40 anos	68 (88,3)	30 (83,3)	0,467
Acima de 40 anos	9 (11,7)	6 (16,7)	
Total	77 (100,0)	36 (100,0)	
**Escolaridade materna**			
Analfabeto/Médio Incompleto	23 (29,9)	10 (27,8)	0,820
Médio Completo/Superior	54 (70,1)	26 (72,2)	
Total	77 (100,0)	36 (100,0)	
**CCEB**			
A/B	55 (71,4)	30 (80,3)	0,172
C/D-E	22 (28,6)	6 (16,7)	
Total	77 (100,0)	36 (100,0)	

Teste Qui-quadrado de Pearson

* valor de p≤0,05

Legenda: N = número de indivíduos; EBAI = Escala Brasileira de Alimentação Infantil; CCEB = Critério de Classificação Socioeconômico Brasil

Em relação à história pregressa, foi observado que entre as crianças com sinais de dificuldades alimentares houve maior proporção das que nasceram pré-termo (p=0,033) quando comparadas com o grupo sem dificuldade. Sobre a introdução alimentar, as crianças que apresentam sinais de dificuldades alimentares, demonstram maior dificuldade no processo de introdução alimentar (p=0,007) do que as demais. Além disso, houve associação entre se alimentar mal até os dois anos e a presença de sinais de dificuldades alimentares (p=0,014). Sobre os sinais sensoriais (Incômodo ao barulho/cheiro/toque), houve maior proporção de crianças com esses sinais entre aquelas que têm sinais de dificuldades alimentares (p=0,001) (Tabela 4).

**Tabela 4:** Análise de associação entre dificuldade para alimentar (EBAI), dados clínicos e percepção da família acerca da alimentação

**Variáveis**	**EBAI**		
	**Sem dificuldade**	**Com dificuldade**	**valor-p**
	**N (%)**	**N (%)**	
**Parto**			
Normal	21 (27,3)	11 (30,6)	0,718
Cesárea	56 (72,7)	25 (69,4)	
Total	77 (100,0)	36 (100,0)	
**Idade gestacional**			
Termo	68 (88,3)	26 (72,2)	0,033*
Pré-termo	9 (11,7)	10 (27,8)	
Total	77 (100,0)	36 (100,0)	
**Amamentação**			
Menos que 6 meses	8 (10,4)	5 (13,9)	0,587
Maior que 6 meses	69 (89,6)	31 (86,1)	
Total	77 (100,0)	36 (100,0)	
**Introdução alimentar**			
Antes 6 meses	33 (42,9)	13 (36,1)	0,496
Após 6 meses	44 (57,1)	23 (63,9)	
Total	77 (100,0)	36 (100,0)	
**Como foi introdução alimentar**			
Fácil	17 (22,1)	17 (47,2)	0,007*
Difícil	60 (77,9)	19 (52,8)	
Total	77 (100,0)	36 (100,0)	
**Alimentação até dois anos**			
Comia bem	68 (88,3)	25 (69,4)	0,014*
Comia mal	9 (11,7)	11 (30,6)	
Total	77 (100,0)	36 (100,0)	
**Incômodo barulho/cheiro/toque**			
Não	61 (80,3)	18 (50,0)	0,001*
Sim	15 (19,7)	18 (50,0)	
Total	77 (100,0)	36 (100,0)	
**Acompanhante durante as refeições**			
Mãe	28 (36,4)	15 (41,7)	0,589
Outros	49 (63,6)	21 (58,3)	
Total	77 (100,0)	36 (100,0)	
**A família se alimenta**			
Junta	50 (64,9)	21 (58,3)	0,499
Separada	27 (35,1)	15 (41,7)	
Total	77 (100,0)	36 (100,0)	
**A criança se alimenta**			
Na mesa com a família	47 (61,0)	22 (61,1)	0,994
Em outro local	30 (39,0)	14 (38,9)	
Total	77 (100,0)	36 (100,0)	
**Criança se alimenta em frente tv/tablet/celular**			
Não	27 (35,1)	8 (22,2)	0,169
Sim	50 (64,9)	28 (77,8)	
Total	77 (100,0)	36 (100,0)	
**Acha prejudicial se alimentar em frente a esses aparelhos**			
Sim, não permito	23 (29,9)	8 (22,2)	0,653
Sim, mas permito para que se alimente bem	46 (59,7)	23 (63,9)	
Não	8 (10,4)	5 (13,9)	
Total	77 (100,0)	36 (100,0)	
**Como considera que seu filho se alimenta hoje**			
Bem	59 (77,6)	23 (63,9)	0,125
Mal	17 (22,4)	13 (36,1)	
Total	76 (100,0)	36 (100,0)	

Teste Qui-quadrado de Pearson

* valor de p≤0,05

Legenda: N = número de indivíduos; EBAI = Escala Brasileira de Alimentação Infantil

Na tabela 5 estão representados os modelos inicial e final da análise multivariada da EBAI com dados sociodemográficos e dados clínicos, por meio da regressão logística binária. Em sua análise observa-se que ficaram no modelo final, com valores significativos, as variáveis idade, com uma razão de chances de 0,32 (68% menor) das crianças com idade entre 4 e 5 anos apresentarem a EBAI alterada quando comparados com as crianças de 2 a 3 anos. Os prematuros apresentarem 3,4 vezes mais chance de alteração na EBAI quando comparados com os que nasceram a termo e 3,7 vezes mais chances de alteração na EBAI entre os que apresentam incômodo com barulho, cheiro ou toque quando comparados com as suas contrapartes.

**Tabela 5:** Análise multivariada de regressão logística binária entre EBAI e idade, dados clínicos e percepção da família acerca da alimentação

**Variáveis**	**EBAI Alterado**					
	**Modelo Inicial**			**Modelo final**		
	**OR**	**IC**	**valor-p**	**OR**	**IC**	**valor-p**
Idade	0,250	0,075-0,829	0,023	0,323	0,078 – 0,693	0,009*
CCEB	0,490	0,135-1,782	0,279	---	---	---
Idade gestacional	3,210	0,847-12,1673	0,086	3,415	1,067 – 10,931	0,039*
Introdução alimentar	0,695	0,209-2,313	0,695	---	---	---
Incômodo barulho/cheiro/toque	2,229	0,675-7,366	0,189	3,743	1,465 – 9,565	0,006*
Alimenta em frente tela	1,621	0,538-4,882	0,391	---	---	---
Como alimenta hoje	1,214	0,397-3,710	0,734	---	---	---
Alimentação até dois anos	1,501	0,350 – 6,440	0,585	---	---	---

Teste de Wald, modelo *Stepwise*

* valor de p≤0,05

Legenda: OR = *Odds ratio*; IC = Intervalo de confiança; EBAI = Escala Brasileira de Alimentação Infantil. **Variáveis de referência:** Idade = 4-5 anos; CCEB = C/D-E; Idade gestacional = pré-termo; Introdução alimentar = difícil; Incômodo barulho/cheiro/toque = sim; alimenta em frente a tela = sim; como alimenta hoje = mal; Alimentação até dois anos = comia mal

## DISCUSSÃO

O presente estudo verificou a associação entre sinais de dificuldade alimentar de crianças de 2 a 5 anos e fatores sociodemográficos, socioeconômicos, história pregressa, escolarização dos pais e percepção da família sobre as dificuldades alimentares da criança. Crianças com idade entre 2 e 3 anos, nascidas pré-termo, e as com incômodo com barulho, cheiro ou ao toque tiveram maiores chances de apresentarem dificuldades alimentares.

Houve prevalência de 31,9% sinais de dificuldade alimentar nas crianças avaliadas, valor que se aproxima de outros estudos descritos na literatura, como um estudo canadense^(15)^ no qual 30% das crianças avaliadas foram caracterizadas como comedores exigentes na faixa etária de dois anos e meio a quatro anos e meio. Outro estudo acompanhou crianças de três a 11 anos e relata que 16 a 22% das crianças apresentavam dificuldades alimentares independentemente da idade e que 39% das crianças foram classificadas como comedores exigentes em algum momento durante o estudo^(16)^.

Em relação à idade, houve tendência das crianças de dois e três anos apresentaram mais dificuldades alimentares do que as demais. Infere-se que isso se deve ao fato de que as crianças nessa faixa etária desenvolvem a autonomia e buscam mais independência no momento da alimentação. Dessa forma, passam a escolher quais alimentos são de sua preferência e a evitar alimentos que são desconhecidos ou que desencadeiam sentimentos de recusa e/ou fobia. Alguns autores relatam que estes comportamentos tendem a diminuir com a idade^(17)^, embora outros estudos relatam que há uma estabilidade na prevalência de dificuldades alimentares entre a faixa etária de dois anos e seis meses até quatro anos e seis meses^(16)^. Em outro estudo, foram realizadas três avaliações: um ano e meio de idade até os seis anos. Foi encontrado que com um ano e meio a prevalência de dificuldades era de 26,5%, aos três anos aumentou para 27,6% e aos seis anos declinou para 13,2%^(18)^, corroborando com outros achados^(16,17)^ que aos três anos há um pico em relação as dificuldades alimentares.

Considerando fatores de risco para dificuldades alimentares na infância, no presente estudo, foi encontrado que crianças prematuras apresentam 3,64 vezes mais chance de desenvolverem sinais de dificuldades alimentares do que crianças que nasceram a termo. Sabe-se que a prematuridade, além de desencadear possíveis dificuldades físicas e psicossociais ao bebê, também propicia maior risco de desenvolver distúrbios de comportamento alimentar^(19-21).^

No estudo de Migraine^(19)^ foram comparadas duas coortes, uma com crianças nascidas a termo e a outra com crianças prematuras. Foi encontrado que as crianças nascidas pré-termo obtiveram pior pontuação no impulso para comer e menor pontuação no repertório alimentar. Outro estudo relata que bebês pré-termo tiveram maior risco de recusa/exigência alimentar^(22)^. Nesses dois estudos, foram incluídos bebês prematuros que apresentam comorbidades associadas, o que difere do atual estudo, que excluiu as comorbidades associadas da amostra.

É importante salientar que a prematuridade é um fator de risco que não deve ser negligenciado mesmo em crianças saudáveis, pois pode estar relacionada à alimentação seletiva e problemas comportamentais durante a refeição, conforme encontrado no presente estudo. Esses comportamentos podem estar relacionados a disfunções orais, ausência ou interrupção precoce de aleitamento materno, introdução alimentar precoce e aspectos neurocomportamentais^(23)^. Além disso, pais de crianças prematuras apresentam grande preocupação e ansiedade em alimentar seus filhos e esses sentimentos podem influenciar de forma negativa o comportamento alimentar no momento da refeição^(22, 24)^.

Outro resultado do presente estudo é a presença de sinais de dificuldades na introdução alimentar e após os dois anos de idade. O processo de introdução de alimentar é bastante desafiador para a família e o conhecimento prévio da família impacta diretamente em como a família vai iniciar a introdução de alimentos e lidar com os desafios durante o processo. A introdução alimentar de forma precoce, antes do sexto mês de vida pode desencadear diversos prejuízos à saúde do bebê, além de influenciar negativamente no processo de aprendizagem do comer, pois o bebê ainda não terá desenvolvido todos os sinais de prontidão alimentar^(25, 26)^.

A literatura relata que um a cada quatro bebês apresentam aversão a introdução de novas texturas e sabores^(4)^, o que vai ao encontro do estudo^(27)^, que descreveu que as crianças caracterizadas como comedores problemáticos, tiveram dificuldades na amamentação e na introdução de sólido e permaneceram até o final da infância. É necessário que o bebê vivencie experiências sensoriais e gustativas na introdução alimentar para ampliação do paladar, a fim de que desenvolva maior repertório alimentar e experiências positivas com a comida^(1)^.

Outro aspecto que influencia significativamente é a oferta repetida de alimentos que foram recusados pelo bebê, que devem ser realizadas de seis a 15 repetições para que a criança consiga aprender a comer determinados alimentos^(12)^. Infere-se que famílias que não receberam informação sobre práticas adequadas podem desencadear comportamentos e experiências negativas nos primeiros anos de vida da criança, que podem permear durante toda a infância.

Não foi encontrada associação entre sinais de dificuldade alimentar e aspectos socioeconômicos dos pais. Contudo, a literatura relata que pais com maior idade, maior nível socioeconômico e mais alto nível de escolaridade apresentam melhor escolha e oferta alimentar^(19, 28)^. Outros estudos relatam que alimentação seletiva foi mais comum em crianças de famílias de baixa renda, e seus pais tendem a ser mais jovens do que os pais de comedores não exigentes^(18)^. Infere-se que fatores sociodemográficos estão relacionados a qualidade do alimento ofertado, porém, de forma isolada, não garante o sucesso no aprendizado do comer. E que outros fatores como: culturais, sociais e comportamentais também influenciam na alimentação.

Segundo a literatura, o ato de alimentar também está diretamente relacionado as entradas multissensoriais do indivíduo e para aqueles com sensibilidades sensoriais, ingerir alimentos com variações de forma, texturas, sabores, cheiros, pode ser aversivo^(29)^. Neste estudo foi encontrado que crianças com sinais de sinais de risco para alterações sensoriais apresentaram mais dificuldade alimentar do que as demais, o que corrobora com estudos que mostram associação entre a capacidade de perceber pequenas mudanças sensoriais nos alimentos e à rejeição alimentar^(17, 23,30)^.

Não foi encontrada associação entre sinais de dificuldades alimentares e a exposição de telas durante as refeições, mas 69% das crianças da amostra se alimentam assistindo a telas. Dentre essas crianças que se alimentam expostas a telas, 61,1% das famílias relatam que acham prejudicial, mas permitem, pois, é a forma de se alimentarem bem. Na literatura, são encontrados estudos que associam o excesso de telas a prejuízos para a saúde infantil, como atrasos na linguagem, dificuldades atencionais, atrasos cognitivos como também, problemas alimentares^(15,25)^.

No estudo de Bahadur^(25)^ observou-se que apenas as crianças com sinais de dificuldades de alimentação apresentavam maior tempo de exposição a telas. Outros estudos relacionam a exposição a TV a distúrbios alimentares e baixo consumo de frutas e vegetais^(15,28)^. Não foram encontrados estudos que relacionam exposição a telas com comportamentos de recusa e seletividade alimentar. Infere-se que as famílias permitem esse hábito para que seus filhos se alimentem em maior quantidade e ingiram alimentos recusados, enquanto estão entretidos nas telas. Sabe-se que o fato da criança se alimentar frente a telas, enquanto estão distraídas, pode afetar a percepção de fome e saciedade regulada pela atenção, além de dificultar a percepção de textura, sabor e consistência, prejudicando o aprendizado do comer^(15,23).^

O presente estudo pode ser considerado inovador em relação ao uso da escala EBAI que foi traduzida e validada recentemente para uso no Brasil. Porém, apresenta algumas limitações que devem ser consideradas. Os questionários utilizados na pesquisa – questionário de caracterização da amostra, CCEB e Escala EBAI – foram preenchidos pelas famílias. Desta forma, os conceitos de dificuldade e normalidade são subjetivos, de acordo com a percepção de cada participante. Além disso, há discrepância entre as idades estudadas, com muitos indivíduos de quatro e cinco anos e poucos de dois e três anos. Dessa forma, se fazem necessários novos estudos com amostra mais homogênea em relação a idade para resultados mais fidedignos e em cenários diferentes.

## CONCLUSÃO

Crianças menores, de dois e três anos, apresentaram maior tendência em apresentar sinais de dificuldades alimentares. Além disso, encontrou-se associação com significância estatística entre prematuridade; dificuldades no processo de introdução alimentar e no segundo ano de vida; e sinais de alterações sensoriais.

Tais resultados fornecem informações que são de extrema importância para profissionais da saúde que atendem bebês e crianças, e podem subsidiar orientações mais específicas e direcionadas as dificuldades alimentares, possibilitando a realização de ações preventivas.
